# Cow’s Milk Allergy: Immunomodulation by Dietary Intervention

**DOI:** 10.3390/nu11061399

**Published:** 2019-06-21

**Authors:** Enza D’Auria, Silvia Salvatore, Elena Pozzi, Cecilia Mantegazza, Marco Ugo Andrea Sartorio, Licia Pensabene, Maria Elisabetta Baldassarre, Massimo Agosti, Yvan Vandenplas, GianVincenzo Zuccotti

**Affiliations:** 1 Department of Pediatrics, Vittore Buzzi Children’s Hospital-University of Milan, 20154 Milan, Italy; elena.pozzi@asst-fbf-sacco.it (E.P.); cecilia.mantegazza@asst-fbf-sacco.it (C.M.); marcoua.sartorio@gmail.com (M.U.A.S.); gianvincenzo.zuccotti@unimi.it (G.Z.); 2 Department of Pediatrics, Ospedale “F. Del Ponte”, University of Insubria, 21100 Varese, Italy; silvia.salvatore@uninsubria.it (S.S.); massimo.agosti@uninsubria.it (M.A.); 3 Department of Medical and Surgical Sciences, Pediatric Unit, University “Magna Graecia” of Catanzaro, 88100 Catanzaro, Italy; pensabene@unicz.it; 4 Neonatology and Neonatal Intensive Care Unit, Department of Biomedical Science and Human Oncology, “Aldo Moro” University of Bari, P.zza Giulio Cesare 11, 70124 Bari, Italy; mariaelisabetta.baldassarre@uniba.it; 5 KidZ Health Castle, Universitair Ziekenhuis Brussel, Vrije Universiteit Brussel, 1090 Brussels, Belgium; yvan.vandenplas@uzbrussel.be

**Keywords:** cow’s milk allergy, immune system, dietary intervention, bioactive peptides, gut microbiota, prebiotics, probiotics

## Abstract

Cow’s milk proteins cause allergic symptoms in 2% to 3% of all infants. In these individuals, the physiological mechanism of tolerance is broken with subsequent possible sensitization to antigens, which can lead eventually to allergic responses. The present review aims to provide an overview of different aspects of immune modulation by dietary intervention in cow’s milk allergy (CMA). It focuses on pathogenetic mechanisms of different CMA related disorders, e.g., gastroesophageal reflux and eosinophilic esophagitis, highlighting the role of dietary management on innate and adaptive immune systems. The traditional dietary management of CMA has greatly changed in the last years, moving from a passive approach, consisting of an elimination diet to relieve symptoms, to a “proactive” one, meaning the possibility to actively modulate the immune system. Thus, new insights into the role of hydrolysates and baked milk in immunomodulation are addressed here. Additionally, nutritional components, such as pre- and probiotics, may target the immune system via microbiota, offering a possible road map for new CMA prevention and treatment strategies.

## 1. Introduction

Cow’s milk allergy (CMA) is one of the most common food allergies in early childhood, with an estimated prevalence of 2% to 3% [1]. A growing body of evidence suggests a close relationship between immunoinflammation and gastrointestinal (GI) motility triggered by dietary antigens [2]. Cow’s milk (CM) free diets and in particular extensive hydrolyzed formulas may reduce gastrointestinal (GI) symptoms due to both immune mechanisms and motility alterations, such as reduced gastric emptying time. Food allergy plays a central role in driving the allergic reaction in eosinophilic esophagitis (EoE) and cow’s milk is the single most common food allergen causing esophageal inflammation [3].

Dietary elimination therapy is thought to target the adaptive immune system, by suppressing antigen-driven T-cell response. Moreover, the role of milk lipids as potential triggers of milk-induced inflammation in EoE is emerging. These findings provide new insights into EoE pathogenetic mechanisms that might change the paradigm of allergy, as a protein antigen-driven response. In the last decade, much has changed in the treatment of food allergy, switching from a passive approach, consisting of a restrictive diet to relieve symptoms, to a “proactive” one, meaning the possibility to actively modulate the immune system.

Protein hydrolysates have been recognized as a potent source of bioactive peptides [4]. They may act locally, e.g., in the gut, by modulating the intestinal microbiota, thereby playing a role in inducing oral tolerance to milk proteins. Additionally, the role of baked milk as a possible form of oral immunotherapy has emerged [5,6]. Maintaining tolerance requires complex interactions between non-immune cells and cells that belong to the gut-associated lymphoid tissue (GALT). Regulatory T cells (Treg) play a crucial role in tolerance. Although different subtypes of Tregs have been identified, the pivotal roles of Foxp3+ in oral tolerance are not completely understood. Gut microbes induce the activation of Tregs, while the same cells are depleted in germ-free mice [7]. Gut microbiota dysbiosis induces alterations in gut function resulting in aberrant Th2 responses towards allergic, rather than tolerogenic response [8]. Therefore, the possibility to actively immunomodulate the immune system targeting microbiota by nutritional factors, e.g., prebiotics and probiotics, represents a novel research strategy. The present review aims to give an overview of the different aspects of immunomodulation by dietary intervention in CMA, based on the most recent evidence.

## 2. Cow’s Milk Allergy and Allergic Dysmotility: A Pleiomorphic Disorder

CMA affects many organs with immediate and delayed reactions [9]. According to the Hill and Hosking classification, CMA may manifest in three different ways: (1) The IgE-sensitized group showing immediate cutaneous reactions and anaphylaxis; (2) the non-IgE-sensitized group with gastrointestinal (GI) symptoms, developing within hours after ingesting moderate amounts of CM; and (3) the group with GI disturbances with or without respiratory symptoms and/or eczema/urticaria, occurring after several hours or days [10].

Allergies may involve the GI tract from mouth to rectum, and may be characterized by an acute (anaphylaxis) or delayed onset [9], the latter including eosinophilic gastroenteropathy, allergic proctocolitis, food protein-induced enterocolitis syndrome, and enteropathy [11]. Allergic dysmotility encompasses different entities, including gastroesophageal reflux disease (GERD), dyspepsia, and constipation, where digestive motility is altered by the neuro-immune-muscle inflammatory interaction triggered by the cow’s milk proteins in predisposed individuals [9,11]. Up to half of the cases of GERD in infants younger than 1 year have been related to CMA based on clinical presentation and improvement on CM [12,13,14]. However, many symptoms, such as weight loss, failure to thrive, food-refusal, irritability, excessive crying, regurgitation, vomiting, anemia, wheezing, and sleep disturbances, may be expressions of both entities [13,14]. By contrast, multiple organ involvement, mucous or bloody stools, an increase in eosinophils blood count, atopic dermatitis, or recurrent bronchitis are more suggestive of CMA [13,14].

However, the reasons for clinical improvement after being started on a milk free diet may vary: The physiological resolution of symptoms over time, an improvement in gastric emptying due to the use of hydrolyzed proteins, or a placebo effect on parental anxiety.

The strongest evidence that intestinal allergic responses can modulate enteric motility originates from a series of studies in animal models. Cytokines production by T helper (Th) 2 cells, and recruitment and activation of either mast cells or eosinophils have been suggested as the major mechanisms potentially linking allergic responses and dysmotility [15]. A Th 2 polarized response determines the release of interleukin (IL)-4 and -13, cytokines that alter motility by upregulating transforming growth factor-beta, with spontaneous contractility of smooth muscle [15]. In a murine model of luminal sensitization, the allergen exposure induced a skew towards a Th2 response with tissue infiltration of IgE degranulating mast cells in the mucosa and mesenteric lymph nodes, causing enteropathy with loose stools and poor weight gain [16]. Moreover, mast cells and their mediators may cause sensorimotor dysfunction of the gut through interactions with the enteric nervous system [17,18,19].

An increase in mast cell density and number in close proximity to submucosal nerve endings has been demonstrated in children with functional dyspepsia and allergies [2]. In allergic children, milk allergen exposure induces rapid degranulation of gastric antral lamina propria mast cells and eosinophils and the release of mast cell tryptase, which interacts with proteinase-activated receptors that colocalize with gastric mucosal nerve fibers. Subsequent electrogastrographic myoelectrical abnormalities occur, determining atopy-related dyspeptic symptoms [2].

In the esophagus, animal studies have shown the degranulation of mast cells and the release of histamine when the mucosa was exposed and injured by acid [20] or when the stress-induced corticotrophin-releasing factor (CRF) signaling system [21,22] was involved. A rise in mast cell numbers and released cytokines has also been demonstrated in humans with non-erosive reflux disease and chest pain syndromes [23,24]. Mast cells play an important role in the esophageal inflammatory reaction and nociception by increasing vagal nociceptive C fibers’ excitability [25,26]. Proteinase activated receptors 2 (PAR2), a target receptor of mast cell derived tryptase, is expressed in epithelial cells, GI smooth muscle cells, and capsaicin-sensitive neurons and regulates GI mucosa barrier functioning and inflammation [27,28,29]. PAR2-mediated pathways have been demonstrated to be important in the pathogenesis of GERD-associated mucosal alterations, such as dilated intercellular spaces and a decrease of tight junction proteins [23,30].

The diagnosis of CMA in patients with GI symptoms is often challenging because of the delayed type of allergic reaction and the absence of specific diagnostic tests: Skin prick or serum specific IgE are usually negative, while atopy patch tests have shown conflicting data [31,32]. Hence, elimination diet followed by an oral open or double blind standardized challenge, in infants or older children is the recommended test to diagnose CMA [33,34]. In allergic patients, GI symptoms disappear in up to 2 to 4 weeks on a CM free diet and relapse when milk is reintroduced [33]. Extensive hydrolisate milk formulas are indicated as the first dietetic choice, whereas elemental formulas should be reserved for more severe cases or eosinophilic disorders [33,35]. A hypoallergenic diet has been proven effective in reducing mast cell mucosal infiltration, thus normalizing immune-nerve interactions and improving motor abnormalities [2,36]. At the same time, hydrolyzed proteins may be effective in these children due to accelerated gastric emptying [13,14]. In patients with persistent symptoms who are on a diet, esophageal pH-impedance may provide data on acid and non-acid reflux exposure and temporal reflux–symptoms association whereas esophagogastroduodenoscopy with esophageal and duodenal biopsies may reveal the presence and the type of esophagitis and/or enteropathy [13].

## 3. Eosinophilic Esophagitis: Insights on Pathogenetic Mechanisms and Dietary Immunomodulation

EoE is a chronic immune-mediated antigen-driven inflammatory disorder characterized by symptoms of esophageal dysfunction and histologic evidence of eosinophilic-predominant inflammation of the esophagus [37,38].

Clinical presentation varies according to age. In infants and younger children, the most common symptoms are food refusal, vomiting, irritability, and failure to thrive. Dysphagia, choking, and food impaction are the most common symptoms in school children and adolescents, as well as in adults [39,40].

Diagnostic criteria for EoE are: (a) Symptoms of esophageal dysfunction; (b) eosinophilic esofageal inflammation, ≥15 eosinophils (Eo)/per high power field (HPF); and (c) exclusion of other causes of esophageal eosinophilia [37,41].

EoE pathogenesis is closely related to atopy. About 70% of patients have a history of atopy, including asthma, IgE-mediated food allergy, allergic rhinitis, and atopic dermatitis. Similarly, about 2/3 of patients have at least one family member with an atopic condition [42]. Peripheral blood eosinophilia is observed in about 50% of patients, and elevated levels of IgE can be detected in 80% of patients. Moreover, up to 80% of patients have positive skin prick tests (SPTs) and/or specific IgE (sIgE) for food or aeroallergens.

Food allergy plays a central role in driving the allergic reaction in EoE, as demonstrated by clinical and histological remission on dietary restriction therapy and exacerbation after food reintroduction [43].

However, the lack of immediate symptoms after food ingestion, the low predictive value of SPTs or SIgE, and the poor response to anti-IgE therapy [44] disprove the hypothesis of a merely IgE- mediated food reaction. The pathogenesis of EoE is most likely a mixed IgE and non-IgE/cell mediated food reaction, in which Th2 cytokines, particularly thymic stromal lymphopoietin (TSLP), interleukin (IL)-4, IL5, IL13, and transforming growth factor-β (TGF-β), and eosinophilic chemokines (eotaxin 1-3/CCL11-CCL24-CCL26 and RANTES/CCL5) play a central role in eosinophilic recruitment, perpetuating local Th2-inflammation. Eosinophils cause tissue damage, remodeling, and fibrosis.

Antigens, primarily food ones, activate the innate and adaptive immune systems, priming the Th2 immune response [45,46].

The goal of therapy is to induce clinical and histological remission (defined as esophageal Eo < 15/HPF). Treatment strategies include drugs (e.g., proton pump inhibitors, corticosteroids) and elimination diets. These therapies both act on esophageal inflammation.

An avoidance diet is thought to target the adaptive immune system, by suppressing antigen-driven T-cell response; it requires elimination of food antigen/s, demonstration of remission, and subsequent sequential reintroduction of each single food in order to identify the causative agent [47,48,49].

Different elimination strategies are currently used in EoE: Elemental diet and empirical elimination diets, such as the six-food groups elimination diet (milk, wheat, soybean/legumes, egg, peanut/nuts, and fish/shellfish) (SFED), four-food elimination diet (milk, wheat, egg, legumes/soy) (FFED), or allergy testing-based food elimination diet (ATBD).

The efficacy of these different dietary treatments ranges from 90.8% for the elemental diet, to 72% for SFED, 55% for FFED, and 45.5% for ATBD [43,50].

After sequential food reintroduction, in the majority of patients (45–85%), one or two causative foods are identifiable. (2) Much evidence supports CM as a major trigger food for EoE. CM is the single most common food allergen causing esophageal inflammation. In both adults and children studies on empiric SFED-FFED or two-food elimination diet (TFED), CM was identified as the single trigger in 18% to 50% of adult patients [3,51,52] and from 30% up to 60% of pediatric patients, in prospective studies [3,52,53].

Moreover, CM elimination diet induced a significant reduction in the mean peak pre- and post-treatment eosinophil count in 68.2% of patients [54,55]. Sensitization to CM (serum sIgE and/or positive skin prick test) was detected in 45.9% of children with EoE, in a large cohort of European EoE children [42].

However, it is known that sIgE correlates poorly with food triggers. Furthermore, it has been observed that CM sIgE levels are paradoxically lower in responders to the CM elimination diet, than in non-responders [56]. These findings are in keeping with the evidence of a non-IgE-mediated reaction.

Nevertheless, even in patients with negative skin prick tests, sIgE to whey protein Bos d 4 (α-lactalbumin) and Bos d 5 (β-lactoglobulin) are frequently detectable by ImmunoCAP assay. Therefore, although IgE response is not the primary mechanism in EoE, Bos d 4 and Bos d 5, minor components of CM, can act as primary antigens for IgE response, triggering T cell-driven inflammation in EoE.

Another antibody isotype currently investigated in EoE is IgG4. IgG4 is an immunoglobulin involved in allergen tolerance and anti-inflammatory activity. High levels of serum and esophageal IgG4 have been found in active EoE in adults [57]. Recently, it has been demonstrated that levels of esophageal IgG4 in EoE patients correlate with the number of esophageal eosinophils, with basal zone hyperplasia, and with levels of IL4 to IL13, and especially IL10, providing evidence that IgG4 correlates with disease activity (i.e., eosinophils and basal zone hyperplasia) and Th 2 inflammation [58]. The highest titers of IgG4 in EoE are to CM and gluten. Levels of serum IgG4 to CM proteins (Bos d 4, Bos d 5 and casein, Bos d 8) are higher in active EoE than in controls. These data suggest a pathogenetic role for IgG4, especially to CM proteins, in EoE.

However, levels decrease on a CM elimination diet not only in subjects with histological remission, but also in subjects without remission, suggesting that IgG4 could be only an epiphenomenon in EoE [59].

Invariant natural killer T cells (iNKTs), a subset of T cells, play a key role in IgE-mediated CM allergy. They are activated by sphingolipids (SLs) rather than by protein antigens. Milk sphingomyelin (milk-SM) activate iNKTs, induce iNKTs’ proliferation, and promote Th2 response [60]. In children with IgE mediated food allergy, especially to CM milk, iNKTs are reduced. Children with active EoE have lower peripheral blood iNKTs with greater Th2 response to milk-SM compared to children with controlled EoE and controls. Esophageal iNKTs are higher in active EoE than in controlled EoE and healthy children. Low peripheral iNKTs could reflect recruitment on site of esophageal inflammation, suggesting a pathogenetic role of iNKTs in EoE [61].

These findings could explain why some foods are more able to trigger EoE than others, and provide new insights into EoE pathogenetic mechanisms. Milk lipids as potential triggers of milk-induced inflammation may change the paradigm of allergy, as a protein antigen-driven response.

## 4. Immune Modulation by Hydrolysate Proteins

Great consideration has recently been given to hydrolysate proteins. Their capacity to reduce allergic symptoms due to the lack of IgE binding epitopes is common knowledge [62]. Therefore, infant formulas containing extensively hydrolyzed proteins are tolerated by allergic infants and are recommended for the management of children with CMA symptoms [63,64].

Furthermore, hydrolysates have been demonstrated as capable of reducing the gut intestinal permeability [65] in ex vivo models. The improved barrier function may decrease the antigen uptake and the antigen contact with the intestinal immune cells in the lamina propria, which may lead to a reduction in allergic symptoms [66].

More recent evidence, however, suggests that hydrolyzed peptides also have an active role in modulating the immune system through different mechanisms both in children with CMA and in those at risk of developing CMA [67,68]

In vitro and ex vivo studies have described hydrolysates as having local and systemic effects on the immune system, including their ability to strengthen the epithelial barrier, via many immunomodulatory mechanisms, such as increasing the regulatory cytokines (e.g., IL-10) or decreasing the inflammatory markers, including cyclo-oxygenase 2 (COX-2), NF-kB, and IL-8, and also by the expression of genes encoding for tight junction proteins [65].

Protein hydrolysates act on the intestinal mesenteric lymph nodes, increasing the number of Treg cells, which are crucial in inducing tolerance [69]. These effects have been demonstrated in murine models analyzing both peptides derived from casein and whey proteins [70,71,72]. Besides enhancing the Treg number in the mesenteric lymphonodes, other effects on the local immune system have been described. In particular, hydrolysates from bovine milk seem to have an anti-inflammatory effect in vivo that is dependent on the protein source (casein or whey). These effects have been observed in animal models after inducing experimental colitis. While pro-inflammatory cytokines, IL-1beta, IL-17, TNF-alpha, and IFN, decreased, an increase in the regulatory cytokine, IL-10, and reduced macroscopical and microscopical damage of the colon mucosa was observed after administration of casein hydrolysate or casein glycomacropeptide [71,73,74]. Based on this and other experimental findings, feeding with a casein eHF is actually recommended as a first-line choice for the management of food protein induced enterocolitis [75].

Feeding with a partially hydrolyzed whey protein diet reduced allergic skin response in a cow’s milk allergy mouse model, by decreasing the levels of Th1, Th17, and enhancing regulatory T and B cells in Peyer plaques after whey challenge [76]. Interestingly, a sequenced peptide derived from whey has been demonstrated to reduce the whey antibody levels in animal models.

Protein hydrolysates also have an effect on the systemic immune system probably via small peptides that pass through the intestinal barrier and enter the systemic circulation. An increase in IL-10 producing regulatory B cells was observed after inducing oral tolerance by administration of intact casein in casein-allergic mice and in the spleen after whey hydrolysates’ administration, respectively [76,77].

**Figure 1:**
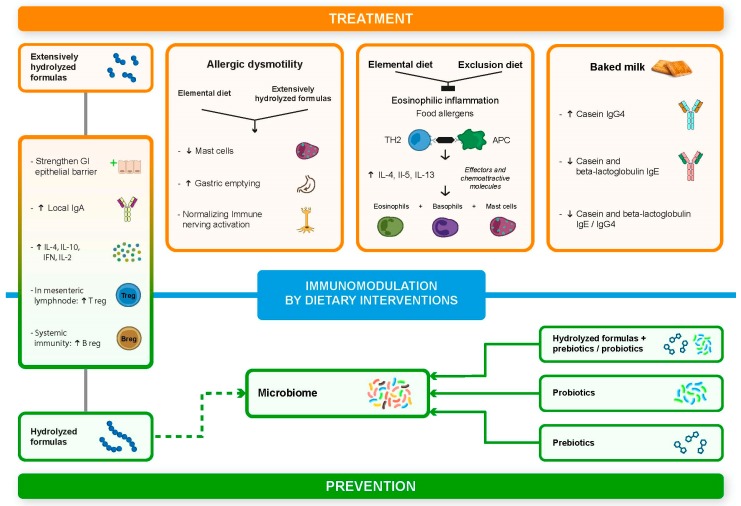
Immunomodulation by dietary interventions. TH2 = T cell helper 2; APC = Antigen Presenting Cell; IL-2 = Interleukin-2; IL-4 = Interleukin-4; IL-5 = Interleukin-5; IL-13 = Interleukin-13; IL-10 = Interleukin-10; IFN = Interferon; T-reg = Regulatory T Cell; B-reg = Regulatory B Cell; ↑ = increase; ↓ = decrease.

Peptides can exert their immune modulatory effects via different mechanisms, among which the direct stimulation of the receptors on immune cells via Toll-like receptors (TLR) is one of the most important [78]. Other mechanisms have been described, including cells’ absorption via transporter or via endocytosis that leads to interactions with inflammatory signaling pathways or conversely to the inhibition of inflammatory signaling pathways [78] (Figure 1).

The majority of studies on the effects of immune modulation by hydrolyzed formula were conducted on ex vivo models; there are very few data regarding how to speed up an increase in tolerance in infants fed with hydrolyzed formulas. However, these studies mostly came from the same group of authors [79,80] and need confirmation before drawing firm conclusions.

The peptides with an immunomodulatory effect are mostly small in size (2 to 20 amino acids), although peptides with a molecular weight over 1000 daltons in whey and soy proteins hydrolysates also seem to have immunomodulatory properties [77,81]. While different protein hydrolysates seem able to directly modulate the local and systemic immune system, the final effect depends on the type of hydrolysate and on the protein source. Furthermore, only few immunomodulatory peptides have been identified up to now. Indeed, further research should be focused on identifying specific immunomodulatory peptides and investigating their immune effects in humans.

## 5. Baked Milk: A Possible Form of Oral Immunotherapy?

Most children with CMA can tolerate baked milk [82,83]. At baseline, children tolerant to baked milk differ from reactive children by having lower milk-specific, beta-lactoglobulin, and casein IgE concentrations [84] and a higher number of Treg cells [85] Although casein IgE levels have been shown to have the best accuracy in predicting the reactivity to a baked milk challenge [86], the test’s performance relies on the decided cut-off points, which, in turn, depend on the sensibility and specificity of the test. Indeed, on an individual basis, an oral food challenge with baked milk should be performed to identify baked milk tolerant subjects as no laboratory testing can predict patient tolerance to baked milk in a reliable and conclusive way.

Many cohort and retrospective studies have hypothesized that CMA resolution occurs more rapidly in cases of regular baked milk assumption [82,84].

However, since cow’s milk tolerance can spontaneously occur in the first years of life, studies without a control group could not explain whether the faster tolerance observed is due to real immune modulation via a baked product, or by a milder phenotype of those patients [82,87,88].

A recent systematic review [88], considering only published observational studies, found weak evidence that the ingestion of baked hens’ eggs or cow’s milk results in an acceleration of tolerance achievement. However, very recently, a controlled randomized clinical trial showed that introducing baked milk in cow’s milk protein allergic patients accelerates the tolerance to fresh milk [5].

It is well known that oral immunotherapy (OIT) plays an immunological role by modulation of humoral and cell immunity. Humoral changes caused by OIT include a decrease in IgE levels and a rise in IgG levels, especially IgG4, which have a protective role on allergic reactions by blocking IgE-mediated basophil and mast cell activation. T cell response modifications include a reduction of Th2 cell line and Th2 cytokines’ expression [89,90]. A study from Goldberg et al. [91] showed that baked-milk reactive patients, who underwent baked milk OIT and reached maintenance dose, present a decrease in IgE reactivity to casein and alpha-lactalbumin. Similar to what happens during OIT [6], studies on the immune profile have suggested that after regular ingestion of baked milk products in baked mild tolerant children, casein IgG4 levels increase [82,84], while casein and beta lactoglobulin-specific IgE levels and casein IgE/IgG4 and beta lactoglobulin IgE/IgG4 ratios decrease [82].

All these findings together suggest that the ingestion of baked milk products could drive a change in immune patterns, speeding up milk tolerance. However, further randomized studies are warranted to confirm this hypothesis.

Besides, the opportunity to reduce the child’s dietary and label-related restrictions has been demonstrated to reduce the stress levels with a beneficial effect on the quality of life of food-allergic children and their families [92,93].

## 6. Gut Microbiota in Perinatal Period and Its Relationship with Immune Function and Allergy Development

Gut microbiota are influenced by several factors occurring during pregnancy and after birth [94,95].

Several studies have evaluated the relationship between bifidobacterial colonization and the development of allergic diseases, including cow’s milk allergy [96,97,98].

Oral feeding determines the major modifications in the composition of intestinal microbiota. Breast milk contains important substances influencing the development of a newborn’s immune system [99]. A recent study [100] suggests that lactoferrin passes throughout breast milk to the intestine of the newborn, promoting the growth of bifidobacteria, which in turn contributes to the regulation of postnatal intestinal development [101].

Human milk contains mainly Lactobacilli and Bifidobacteria with an estimated number of ingested bacteria of 1 × 10^5^ to 1 × 10^7^ per 800 mL of milk consumed daily [102]. These bacteria stimulate endogenous production of secretory IgA [103], activation of T regulatory cells [104], and anti-inflammation response [105,106]. Gut microbiota establishment in early life is crucial for the success of oral tolerance, mediated by Foxp3þ and Treg, known to inhibit immune activation [107,108]. Germ free mice showed a Th2-skewed response [109]. An early exposure to pro-and/or prebiotics during the prenatal period and in early life might be beneficial in preventing Th2- mediated allergic disease, including food allergy [110].

## 7. Prebiotics and CMA

An increasing body of evidence shows that the gut microbiota contributes to the maturation of the immune system [111]. An altered patterns of early colonization, e.g., “dysbiosis,” predisposes people to allergic diseases. Prospective studies have demonstrated that a gut microbial imbalance due to reduced diversity in the early years of life is associated with an increased risk of developing food sensitization and atopic eczema [112,113,114]. Although the specific microbiota dysfunction in allergies remains unclear, both prebiotics and probiotics probably modulate immune development through a number of different pathways that can be modified by host and environmental factors. Prebiotic carbohydrates are a major substrate for bacterial growth, and stimulate selectively the growth and/or activity of beneficial species of the gut microbiota. The bifidogenic effect of human milk (a rich source of oligosaccharides) and of certain prebiotics (i.e., fructo- and galacto-oligosaccharides) added to infant milk formulas has long been reported [115,116].

Human milk oligosaccharides (HMOs) may both reduce the adhesion of pathogens and act as metabolic substrates for select species, contributing to the shaping of the infant gut microbiota and modulating the immune system [117] and health of infants [118]. However, there are hundreds of different HMOs, with specific properties and functions [97]. Up to now, only a small number of HMOs have been synthetized and added to infant formula, showing beneficial results [97]. According to a recent study, infants fed with human milk containing low Lacto-N-fucopentaose III (LNFP) concentrations were more likely to become affected by CMA compared to infants receiving high LNFP III-containing milk (odds ratio 6.7, 95% CI 2.0–22) [119].

A systematic review [120] on HMOs reported a protective effect against CMA by 18 months of age.

A beneficial effect of a special mixture of prebiotics (short-chain galacto- and long chain fructo-oligosaccharides) on the development of atopic dermatitis in a high risk population of infants was shown for the first time in 2006 [116].

Fewer infants in the intervention group (hydrolyzed protein formula + prebiotic mixture) developed atopic dermatitis compared to infants in the control group (hydrolyzed protein formula + maltodextrine) (9.8%; 95 CI 5.4–17.1% vs. 23.1%; 95 CI 16.0%–32.1%) In the intervention group, a significantly higher number of faecal bifidobacteria was detected compared to the controls [116]. A systematic review and meta-analysis found no effect on the onset of asthma whilst it did find a significant reduction in eczema (four studies, 1218 infants; risk ratio (RR) 0.68, 95% CI 0.48–0.97, with a number needed to treat 25 (95% CI 14 to >100)) [121]. Conversely, a more recent systematic review reported no difference in eczema (RR: 0.57, 95% CI: 0.30e1.08). Only one study evaluated the risk of food allergy and found a reduced risk (RR: 0.28, 95% CI 0.08e1.00) in prebiotic-treated infants [122].

A very recent study [111] assessed the effect of a partially hydrolyzed protein formula supplemented with non-digestible oligosaccharides on the prevention of eczema in 138 infants at high risk of allergy. Infants receiving the prebiotic formula had a fecal microbial composition, metabolites, and pH closer to that of breast-fed infants than that of infants receiving standard cow’s milk formula. Infants with eczema by 18 months showed decreased acquisition of *Eubacterium* and *Anaerostipes* species with increased lactate and reduced butyrate levels [111].

A similar effect was also shown in non-at-risk infants [123]: In total, 414 infants received an intact protein formula containing a specific mixture of neutral oligosaccharides and pectin-derived acidic oligosaccharides compared to 416 infants fed with a control formula without oligosaccharides. Up to the first year of life, atopic dermatitis occurred in a significantly higher number of infants from the control group (9.7%) than the prebiotic group (5.7%) [123]. The addition of lactose to an extensively hydrolyzed formula significantly increased the total fecal counts of *Bifidobacteria* and lactic acid bacteria, and decreased that of *Bacteroides*/*Clostridia*.s. Moreover, lactose significantly increased the concentration of total short-chain fatty acids, especially acetic and butyric acids, as demonstrated by the metabolomic analysis [124].

A recent multicenter double-blind randomized controlled trial [125] investigated the effects of an amino acid-based formula (AAF), including fructo-oligosaccharides, and the probiotic strain, *Bifidobacterium* breve M-16V, in 35 infants with suspected non-IgE-mediated CMA. After 8 weeks of diet, the median percentage of *Bifidobacteria* was significantly (*p* < 0.001) higher in the test group than in the 36 control subjects fed non-supplemented AAF (35.4% vs. 9.7%), whereas *Eubacterium* rectale/*Clostridium coccoides* group in feces was lower (9.5% vs. 24.2%) and similar to that detected in breastfed infants (55% and 6.5%, respectively).

A subsequent double-blind randomized controlled multicenter trial with the same study groups and formulas confirmed the same fecal microbiota changes at 26 weeks [126]. Safety parameters were similar between groups.

Data from animal models have shown that in whey-sensitized mice, dietary supplementation with short chain galacto-oligosaccharides, long chain fructo-olgosaccharides, pectin-derived acidic oligosaccharides, and/or mixtures of the above prebiotics effectively reduced allergic symptoms but differentially affected mucosal immune activation. In whey-sensitized mice, mixtures of prebiotics increased the number of Foxp3+ cells in the proximal small intestine compared to sham-sensitized mice [127]. The increased expression of Th2 and Th17 mRNA markers in the small intestine of whey-sensitized mice was prevented by the mixture of galacto and fructo-oligosaccharides. Adding pectin-derived acidic oligosaccharides to this mixture enhanced Tbet (Th1), IL-10, and TGF-β mRNA expression, which was maintained in the distal small intestine and/or colon [127]. Interestingly, a more recent study [128] showed that co-administration of oligosaccharides and partially hydrolyzed whey protein can induce immunological tolerance in mice orally sensitized with whey and/or cholera toxin on day 35, particularly if the intake was on a daily basis. The oligosaccharide composition seems to influence the tolerance inducing mechanisms and was associated with the decrease of *Lactobacillus* species, being replaced by Bacteroidales family S24-7 members and with the relative abundance of Prevotella [128].

In 2011, the European Society for Paediatric Gastroenterology Hepatology and Nutrition (ESPGHAN) Committee on Nutrition concluded that there was insufficient evidence to recommend the use of prebiotics in infant formula to prevent atopic disease [129].

Conversely, based on the Grading of Recommendations Assessment, Development and Evaluation (GRADE) approach, in 2016, the World Allergy Organization guideline panel suggested the use of prebiotic supplementation in not-exclusively breastfed infants; however, both recommendations were based on a very low certainty of evidence [122].

At present, despite some promising results mainly related to the effect of specific prebiotics on the gut microbiota, clinical evidence of the beneficial effect of prebiotics in CMA is still inconclusive [128].

## 8. Probiotics and CM

Several studies have shown that probiotic supplementation given to women during pregnancy and lactation can modulate the microbial milk composition and immunity-modulating molecules, with health benefits ranging from gastrointestinal symptoms to allergies, transferred to the newborn [130]. Administration to mothers of a probiotic mixture (sold in continental Europe and the USA as Vivomixx^®^ and Visbiome^®^-, -Danisco-Dupont, WI, USA,) resulted in an increase of Lactobacilli and Bifidobacteria in both colostrum and mature milk [131] in the “probiotic group” with respect to the “placebo group”, and in breast milk concentrations of secretory IgA and TGF-β and IL-10 (anti-inflammatory and immunomodulatory cytokines) [132]. This increasing gut maturation influences a newborn’s IgA production and seems to improve gastrointestinal functional symptoms in infants [132]. TGF-β ingested through breast milk restrains inflammatory responses in intestinal epithelial cells and T cells and exerts a modulation on the immune tolerance towards dietary antigens and indigenous intestinal microbes by induction of Treg cells [132]. It also increases the IgA production in newborns, improving the intestinal barrier function [133].

Maternal probiotic supplementation during pregnancy and breastfeeding seems to prevent atopic eczema in children [130]. The results of the main studies (RCT) are shown in Table 1 [134,135,136,137,138,139,140,141] (Table 1).

Further studies are requested in order to confirm the possibility of preventing other allergic disorders with perinatal probiotic administration.

The World Allergy Organization (WAO) [142] recommends the use of probiotics in pregnant and breastfeeding women and in non-exclusively breastfed infants at high risk of allergic disease. On the other hand, the Academy of Allergy and Clinical Immunology [143] and European Society for Paediatric Gastroenterology, Hepatology, and Nutrition [129] do not recommend the use of probiotics and/or prebiotics for the prevention of allergic diseases. However, the WAO guideline panel recognizes that the recommendations of both probiotics and prebiotics are conditional and based on very low quality evidence.

In terms of the therapeutic property of probiotics, it has been demonstrated [144] that in infants with proctocolitis, the addition of *Lactobacillus rhamnosus* LGG to an extensively hydrolyzed cow’s milk protein formula determines a greater decrease in fecal calprotectin [145] and a reduction in the number of infants with a persistence of occult blood in stools after 1 month. LGG could enhance the intestinal mucosa’s barrier function and participate in the degradation of protein antigens, compete with pathogenic bacteria, and promote early immune system maturation towards non-allergy. A recent systematic review considered a randomized trial, involving 895 pediatric patients with CMA. The primary outcome of interest was relief of symptoms in terms of a reduction of the severity of atopic dermatitis (measured by the SCORing Atopic Dermatitis (SCORAD) index). Overall, a decrease of the SCORAD index was shown in subjects given probiotics, but the results were imprecise and do not permit firm conclusions to be drawn [146].

The results of Randomized Controlled Trials (RCT) on probiotics use in CMA treatment are shown in Table 2 (Table 2).

Great interest has recently arisen regarding the possible role of probiotics administration in fasting tolerance. Despite some evidence that a specific strain, such as *Lactobacillus rhamnosus*, LGG administration may induce tolerance among infants with CMA with a long-lasting effect [147]. Although, no general conclusions can be drawn, due to inconclusive evidence and imprecise results [146]. Further studies are required to investigate the effects of pre- and postnatal probiotic supplementation on the development of systemic and mucosal immunity. Similarly, the most effective strains, dosages, or optimal duration of treatment still need to be defined.

The use of probiotics is in general safe during pregnancy and in newborns (see Table 1). Kuitunen et al. [153] reported that newborns supplemented with probiotics before and after birth had significantly lower hemoglobin levels compared to the placebo group at six months of life. This effect was considered to be transient

Without proper identification of the strains the clinical evidence regarding one product could not be transferred from one product to another. This is the reason why the limiting of information to probiotic genera/species is not the best choice [154]. Without consideration of current regulatory and commercial loopholes, assessing harm will be difficult for researchers, physicians, and patients. More stringent regulations mandating full disclosure of the probiotic microorganisms at the strain level and the origin of the product and manufacturing changes are a prerequisite for proper safety and efficacy reporting [155].

## 9. Conclusions

Much has changed in recent years in food allergy management, moving from a one-size approach to a personalized one, associated with the specific food allergy phenotype. While different protein hydrolysates seem able to modulate the immune system, the few in vivo data, although promising, do not allow us to draw conclusions on their effect on tolerance achievement. Furthermore, the paucity and heterogeneity among the studies currently limit one’s ability to compare the results and to recommend the routine use of prebiotics and probiotics for prevention and treatment of CMA.

## Figures and Tables

**Table 1:** Probiotics administration during pregnancy and breastfeeding for the prevention of allergic disorders.

Author, Year	Study	Subjects	Strain, Dose, Beginning of the Treatment (S), End of the Treatment (E)	Placebo	Outcomes	Follow-Up (Years)	Side Effects
Dotterud et al. [134]	RCT	415 pregnant women	LGG 5 × 10^10^ CFU, Bb-12 5 × 10^10^ CFU and La-5. 5 × 10^9^ (CFU) daily S: 4 weeks before expected delivery date E: 3 weeks after delivery (breastfeeding)	yes	Probiotic supplementation reduces incidence of atopic dermatitis (AD) in children	2	No
Enomoto et al. [135]	Open-trial	166 pregnant women and newborns	BB536 5 × 10^9^ CFU and BB M-16V 5 × 10^9^ CFU daily S: 4 weeks before expected delivery date E: 6 months after delivery (to infants)	no	Probiotic supplementation reduces incidence of AD in children	3	no
Wickens et al. [141]	RCT	423 pregnant women	LR HN001 6 × 10^9^ CFU daily S: from 14–16 weeks gestation E: 6 months after delivery (breastfeeding)	yes	Probiotic supplementation does not prevent AD in infants	1	no
Ou et al. [138]	RCT	191 pregnant women and related newborns	LGG ATCC 53103, 1 × 10^10^ CFU daily S: From the second trimester of pregnancy; E: 6 months after delivery (to mothers and infants) during breastfeeding	yes	Probiotic supplementation doesn’t prevent infant allergic disease (AD, allergic rhinitis, asthma)	3	no
Rautava et al. [139]	RCT	241 pregnant women	LPR 1 × 10^9^ CFU BL999 1 × 10^9^ CFU ST11 1 × 10^9^ CFU daily S: 2 months before expected delivery E: 2 months after delivery (breast-feeding)	yes	Probiotic supplementation prevents infant eczema	2	Not observed
Kim et al. [136]	Randomized placebo-controlled trial	112 pregnant women and newborns	BGN4 1.6 × 10^9^ CFU, AD011 1.6 × 10^9^ CFU, and AD031 1.6 × 10^9^ CFU daily S: 4–8 weeks before expected delivery E: 6 months after delivery (to mothers during breastfeeding and to infants)	yes	Probiotics supplementation reduces incidence of AD in children	1	yes
Niers et al. [137]	Double-blind, randomized, placebo-controlled trial	136 pregnant women and newborns	BB: 1 × 10^9^ CFU; BL 1 × 10^9^ CFU; LL 1 × 10^9^ CFU S: last 6 weeks of pregnancy E: 12 months after delivery (to infants)	yes	Probiotics supplementation reduces the incidence of AD in children at 3 months of life	24 months after delivery	no
Simpson et al. [140]	Randomized placebo-controlled trial	415 pregnant women	Probiotic milk: LGG, 5 × 10^10^ CFU; La-5 5 × 10^9^ CFU and Bb-12 5 × 10^10^ CFU S: from 36 weeks gestation E: 3 months after delivery (breastfeeding)	yes	Probiotics supplementation reduces incidence of AD	6 years after delivery	no

LGG: *Lactobacillus rhamnosus* GG; Bb-12: *Bifidobacterium animalis subsp. Lactis* Bb-12; La-5: *L. acidophilus* La-5; CFU: colony-forming unit; BB536: *B. longum* BB536 [ATCC BAA-999]; BB M-16V: *B. breve* M-16V [LMG 23729]; LR HN001: *Lactobacillus Rhamnosus* HN001; LG *LPR: Lactobacillus rhamnosus LPR*; *BL999: Bifidobacterium longum BL999*. *ST11: L paracasei ST11*; BGN4: *Bifidobacterium bifidum* BGN4; AD011: *Bifidobacterium lactis* AD011; AD031: *Lactobacillus acidophilus* AD031; BB: *Bifidobacterium bifidum*; BL: *Bifidobacterium lactis*; LL: *Lactococcus lactis*; AD: Atopic Dermatitis.

**Table 2:** Probiotics in cow’s milk allergy CMA treatment.

Author, Year	Study Design	Subjects	Strain, Dose (D)	Placebo	Outcomes	Treatment Period (Months)	Side Effects
Baldassarre et al. [144]	RCT	30 infants	LGG 1 × 10^6^ CFU/g	yes	Probiotic supplementation improves gastrointestinal symptoms (hematochezia and fecal calprotectin)	1	No
Berni Canani et al. [79]	RCT	80 infants	LGG, 1.4 × 10^7^ CFU/100 mL	yes	Probiotic supplementation accelerates tolerance acquisition to cow’s milk proteins	12	No
Berni Canani et al. [80]	RCT	260 infants	LGG (dose not specified)	yes	Probiotic supplementation accelerates tolerance acquisition to cow’s milk proteins	12	No
Berni Canani et al. [147]	RCT	220 children	LGG (dose not specified)	yes	Probiotic supplementation reduces the incidence of other allergic manifestations and hastens the development of oral tolerance to cow’s milk proteins	36	No
Dupont et al. [148]	RCT	119 infants	LC CRL431 and Bb-12 (dose not specified)	yes	Probiotic supplementation significantly improves the SCORAD index and growth indices	6	No
Hol et al. [149]	RCT	119 infants	LC CRL431 and Bb-12 1 × 10^7^ CFU/g formula	yes	Probiotic supplementation does not accelerate tolerance acquisition to cow’s milk proteins	6	No
Kirjavainen et al. [150]	RCT	35 infants	LGG ATCC 53103 1 × 10^9^ CFU/g	yes	Supplementation with viable probiotics improves the SCORAD index	2	Diarrhea (with heat-inactivated LGG)
Majamaa et al. [151]	RCT	31 infants	LGG ATCC 53103- 5 × 10^8^ CFU/g formula twice a day	yes	Probiotic supplementation improves the SCORAD index and reduces markers of intestinal inflammation	1	No
Viljanen et al. [152]	RCT	230 infants	LGG (ATCC 53103) 5 × 10^9^ CFU vs. LGG 5 × 10^9^ CFU, LR LC705- 5 × 10^9^ CFU, Bbi99- 2 × 10^8^ CFU, and PJS- 2 × 10^9^ CFU twice a day	yes	Probiotic supplementation improves the SCORAD index in IgE-sensitized infants but not in non-IgE-sensitized infants	1	No

LGG: *Lactobacillus rhamnosus* LGG; CFU: colony-forming unit; LC CRL431: *L. casei* CRL431; Bb12: *B. lactis* Bb-12 (*B animalis* subspecies lactis); LR LC705: *L. Rhamnosus* LC705 Bbi99: *Bifidobacterium breve* Bbi99; PJS: *Propionibacterium* JS.
